# Walking but Not Barking Improves Verb Recovery: Implications for Action Observation Treatment in Aphasia Rehabilitation

**DOI:** 10.1371/journal.pone.0038610

**Published:** 2012-06-13

**Authors:** Paola Marangolo, Susanna Cipollari, Valentina Fiori, Carmela Razzano, Carlo Caltagirone

**Affiliations:** 1 Facoltà di Medicina, Università Politecnica Marche, Ancona, Italy; 2 IRCCS Fondazione Santa Lucia, Roma, Italy; 3 Università di Roma, Tor Vergata, Roma, Italy; University of Bologna, Italy

## Abstract

Recent studies have shown that action observation treatment without concomitant verbal cue has a positive impact on the recovery of verb retrieval deficits in aphasic patients. In agreement with an embodied cognition viewpoint, a hypothesis has been advanced that gestures and language form a single communication system and words whose retrieval is facilitated by gestures are semantically represented through sensory-motor features. However, it is still an open question as to what extent this treatment approach works. Results from the recovery of motor deficits have suggested that action observation promotes motor recovery only for actions that are part of the motor repertoire of the observer. The aim of the present experiment was to further investigate the role of action observation treatment in verb recovery. In particular, we contrasted the effects induced by observing human actions (e.g. dancing, kicking, pointing, eating) versus non human actions (e.g. barking, printing). Seven chronic aphasic patients with a selective deficit in verb retrieval underwent an intensive rehabilitation training that included five daily sessions over two consecutive weeks. Each subject was asked to carefully observe 115 video-clips of actions, one at a time and, after observing them, they had to produce the corresponding verb. Two groups of actions were randomly presented: humans versus nonhuman actions. In all patients, significant improvement in verb retrieval was found only by observing video-clips of human actions. Moreover, follow-up testing revealed long-term verb recovery that was still present two months after the two treatments had ended. In support of the multimodal concept representation's proposal, we suggest that just the observation of actions pertaining to the human motor repertoire is an effective rehabilitation approach for verb recovery.

## Introduction

It is well known that in aphasic patients word-finding difficulties are the most pervasive symptom of language breakdown. Different rehabilitation therapies based either on the simple use of gesture [1]–[3] or on gestures paired with verbal production [4]–[8] have been proposed. In a work by Hanlon et al. [1], the effect of different unilateral gestural movements on naming to confrontation were examined. Results showed that activating the hemiplegic right arm to execute a communicative but non-representational pointing gesture facilitated aphasics' naming abilities. Raimer et al. [6] examined the effect of gestural treatments using pantomimes paired with verbal training for noun and verb retrieval in a group of aphasic patients. Results showed a specific improvement in naming trained nouns and verbs but not in untrained words.

According to these data, gestures and speech are two separate domains. Gestures might interact either at an early stage, when the message to be conveyed is being prepared for linguistic formulation (“conceptual gestures”) or during later stages, when the retrieval of lexical items momentary fails (“lexical gestures”) [1], [9]–[10].

More recently, a different interpretation has been proposed [11]. In agreement with an embodied cognition viewpoint [12]–[13], some authors have suggested that gesture and speech are strongly connected to the same conceptual representation. Words whose retrieval is facilitated by gestures are more likely to be analogically encoded in a multimodal representation including sensory-motor features [11], [14]. The more a word is grounded in sensory-motor features, the more the real execution of the corresponding gesture will facilitate its retrieval [11]. Very recently, Marangolo et al. [15] presented data in favour of the embodied representation proposal, which did not confirm that intentionally performing a gesture prior to name [11] is a necessary prerequisite for enhancing naming. The authors [15] investigated whether the “observation of semantically congruent actions” and/or “the observation and execution of semantically congruent actions” would improve verb-finding difficulties in a group of six aphasic patients. Differently from most of the previous reports [4]–[8], neither treatment was combined with verbal cues. Results showed a significant improvement in verb retrieval not only when subjects, prior to naming, were required to observe and then to execute the action performed by the examiner (“action observation and execution”), but also when they were required to simply observe the action (“action observation”). In both conditions, this improvement was still present two months after the two treatments ended. No significant effects were found in the control condition in which patients first observed the action performed by the examiner and then had to execute a meaningless movement. In short, results clearly showed that the simple observation of a semantically congruent action has reinforced verb retrieval in the same way as the actual execution of the action. Current views assume that a shared motor representation for the execution and observation of actions, the so-called “Mirror Neuron System”, is implicated in understanding others' actions by means of an automatic matching process that links observed and performed actions [16]–[20] In line with the Mirror Neuron hypothesis and the multimodal concept representation proposal [14], the authors argued that in their work the observation of the performed action was sufficient to activate in the semantic system its corresponding sensory-motor representation, which served as input at the lexical level facilitating verb retrieval [15].

The role of action observation, as an effective strategy in neurorehabilitation, has been yet supported by several recent studies showing that action observation has a positive impact on recovery of motor deficits after stroke [21]–[24].

Ertelt et al. [22] combined observation of daily actions with concomitant physical training of the observed actions in eight stroke patients with moderate, chronic motor deficit of the upper limb. A control group of eight participants post-stroke paired motor training with observation of geometric symbols and letters. Significant functional improvement on standard scales occurred for combined action observation and motor training compared with controls despite a stable pre-training baseline. Very recently, these results were replicated in a larger group of twenty-eight participants with chronic upper limb motor deficits, a group affected by Parkinson's disease [21], [23] and post-surgical orthopedic patients [24].

However, it is still an open question as to what extent the Action Observation approach is really effective. It has been suggested that the Mirror Neuron System is indeed more strongly activated by actions that can be reproduced and are part of the motor repertoire of the observer. In an fMRI study, Buccino et al. [25] investigated whether the observation of actions performed by nonconspecifics (e.g. monkey and dog) would activate the same cortical areas that are active when subjects observe actions made by humans. Results showed that when the observed action is common to animals and humans (e.g. biting), there is a clear overlap between the activated areas. In contrast, during the observation of an action that does not belong to humans (e.g. barking), there was a clear difference in the distribution and extent of activations. Similar results were obtained comparing familiar versus unfamiliar actions [26]–[28] and human agents versus robotic agents [29]–[30], even when all actions were matched for kinematics [30]–[32]. Actions that are not part of the motor repertoire of the observer and which therefore cannot be reproduced appear to be recognized in non motor terms.

To further evaluate the proposal that action observation might be an effective rehabilitation approach in stroke patients [15], [21]–[24], in this study we wanted to further investigate as to what extent action observation might be a useful tool in language rehabilitation. In particular, we contrasted the effects induced by observing human video-clips of actions (e.g., dancing, biting, pointing, kicking) versus the results obtained by observing non human video-clips of actions (e.g., barking, printing) in seven patients with lexical verb retrieval disturbances.

## Materials and Methods

### Participants

Seven chronic aphasic patients (5 females and 2 males) classified as right-handed according to the Edinburgh Inventory [33] were included in the study. Inclusion criteria were the presence of a single left cerebrovascular accident (CVA) at least six months prior to the investigation (see Table 1) with no previous neurological, psychiatric, or substance abuse history. All were native Italian speakers.

**Table 1:** Sociodemographic and Clinical data of the seven aphasic subjects.

Participants	Sex	Age	Educational Level	Type of Aphasia	Time post-onset	Verb naming (BADA)	Verb Comprehension (BADA)	Token test
1	F	53	11	Non fluent	1 year, 5 months	15/28	20/20	17/36
2	F	64	13	Non fluent	3 years, 2 months	14/28	20/20	18/36
3	M	60	11	Non fluent	4 years	1/28	20/20	10/36
4	F	53	13	Non fluent	11 months	18/28	20/20	16/36
5	M	43	13	Non fluent	6 years, 6 months	12/28	20/20	16/36
6	F	54	13	Non fluent	6 years, 7 months	11/28	20/20	12/36
7	F	57	12	Non fluent	10 years, 10 months	14/28	20/20	17/36

For each test, the number of correct responses are reported.

All patients were classified as nonfluent aphasics because of their reduced spontaneous speech with short sentences and frequent word-finding difficulties. They had no articulatory deficits with preserved word repetition. In a task requiring the ability to match an auditory presented verb to one of the two semantically related pictures (Verb Comprehension task), their comprehension was intact. As regards commands and auditory sentences, their comprehension was still severely (patient 3 and 6) to mildly impaired (patient 1, 2, 4, 5, 7; 29/36 cut-off score, Token test) [34]. In a naming task, all patients had verb retrieval deficits (the Battery for the analysis of aphasic disorders, BADA test) [35] (see Table 1). On the ideative, ideomotor, bucco-facial tests and on the Gait Apraxia test, no patient revealed an apraxia disorder [36]–[37].

### Ethics Statement

The Institutional Review Board of the IRCCS Fondazione Santa Lucia, Rome, Italy specifically approved this study. The data analyzed in the current study were collected in accordance with the Helsinki Declaration and our institutional review board.

### Materials

Before the training, a list of 115 human (N = 78) and non human (N = 37) videotaped actions matched for frequency of use and length were selected (human actions mean frequency  = 23, SEM  = 3; non human actions mean frequency  = 22; SEM  = 3, t = 0.21, p = .83; human action mean length  = 8, SEM  = 0.2; non human actions: mean length  = 8, SEM  = 0.2; t = 0.68, p = .50). Human actions were divided into four categories depending on the motor effector performing the action (hand action (N = 21), e.g., cutting; mouth action (N = 20), e.g., drinking; foot action (N = 16), e.g., kicking; body action (N = 21), e.g., dancing). Non human actions were divided into two categories of natural (N = 14, e.g., barking, raining) and mechanical actions (N = 23, e.g., printing, digging). All categories were matched for length and frequency of use (for all t-test comparison → p =  n.s.).

To measure the patient's response consistency to each item, the 115 videotaped actions were presented to the patients on a desktop once a day for three consecutive days and they had to respond within 15 seconds. The actions which were correctly named three times out of three would be excluded from rehabilitation. Neither verb was correctly named three times out of three but all patients gave inconsistent responses to all stimuli either producing an incorrect answer or an omission. Therefore, for each patient, all the 115 items were selected for the training.

### Treatment procedure

Each participant was asked to participate in an intensive language training which included five daily sessions over two consecutive weeks. In each session, each patient was asked to carefully observe the 115 video-clips of actions projected, one at a time, on a desktop with a 100×200 cm screen. Each action remained on the screen for 15 seconds. After observing the action, he/she was asked to produce the corresponding name.

During the session, the therapist did not facilitate the patient with verbal cues but simply reminded him/her to pay attention to the video clips and manually recorded the answers. If the patient failed to produce an answer or produce an incorrect verb, after 15 seconds the therapist presented the subsequent action. Over sessions, all actions were randomly presented to the subject. The treatment was carried out in a quiet room with the patient sitting comfortably in front of the screen.

At the end of the treatment, to control for a possible transfer of rehabilitation effects also in other tasks, all patients were again administered the language examination tasks.

### Data Analysis

The subjects' performance was evaluated by taking into account the mean percentage of accuracy rates (number of stimuli correctly named divided by the number of stimuli presented in each block). First, pre-treatment (baseline performance) and post-treatment mean percentage of correct responses (after two weeks) on the 115 videotaped actions were compared within group by using a 2×2 repeated-measures ANOVA. Two within-subject factors were included: *Time* (two levels, baseline (T1) vs. end of treatment (T10)) and *Condition* (two levels, human vs. non-human actions). Interaction was explored using the Scheffè post-hoc test.

In order to control for differences in the mean percentage of correct responses between categories within each group of actions (human and non human), two separate repeated-measures ANOVAs were also performed. In each analysis, two within-subject factors were included: *Time* (two levels, baseline (T1) vs. end of treatment (T10)) and *Condition* (four levels: mouth, feet, body and hand category for human actions; and, two levels: natural and mechanical category for non human actions). Interaction was explored using the Scheffè post-hoc test.

In order to measure long-lasting beneficial effects, a repeated-measures ANOVA was also run on three follow-up sessions carried out at one week, one month and two months after the end of the treatment. Two within-subject factors were included: *End-Post Treatment* (four levels, tenth day vs. first follow-up vs. second follow-up vs. third follow-up) and *Condition* (two levels, human vs. non-human actions). Interaction was explored using the Scheffè post-hoc test.

Finally, to regard for a possible transfer of verb treatment effects in the language examination, different chi square tests were performed to compare the patient's percentage of correct responses before and after the treatment in the verb naming and in the description task of the BADA examination [35].

## Results

The analysis showed a significant effect of *Time* [two levels, baseline (T1) vs. end of treatment (T10), F _(1,6)_ = 31.77; p = .00) and *Condition* (two levels, human vs. non-human, F _(1, 6)_ = 63.28; p = .00). Subjects' performance significantly improved at the end of training with respect to baseline performance (mean  = 51%, SEM  = 7 (T10) vs. mean  = 33%, SEM  = 5 (T1), p = .0001). Moreover, the mean percentage of correct responses for human actions was significantly greater than for non-human actions (mean  = 51%, SEM  = 7 (human actions) vs. mean  = 33%, SEM  = 5 (non human actions) p = .000). The interaction of *Time × Condition* (F _(1,6)_ = 54.83; p = .00) was also significant.

**Figure 1:**
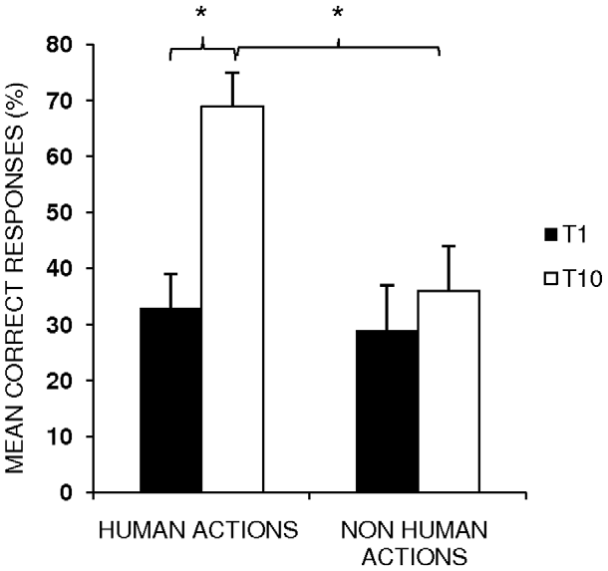
Mean percentage of correct responses for human and non human actions at T1 and T10, respectively (*p = .000). Error bars represent standard error of the mean.

The Scheffè post-hoc test revealed that, while the mean percentage of correct responses for non human actions at the end of training did not significantly differ from baseline performance (mean  = 33%, SEM  = 6 (T10), vs. mean  = 29%, SEM  = 6 (T1), p = .23), a significant improvement was observed between the end of treatment and baseline performance for human actions (mean  = 69%, SEM  = 8 (T10) vs. mean  = 36%, SEM  = 8 (T1), p = .000). Moreover, while no significant differences emerged in the mean percentage of correct responses between human and non human actions at baseline (mean  = 33%, SEM  = 6 (human actions) vs. mean  = 29, SEM  = 6 (non human actions), p = .6), the mean percentage of response accuracy was greater for human actions than for non human actions at the end of training (mean  = 69 %, SEM  = 8 (human actions) vs. mean  = 36%, SEM  = 8 (non human actions), p = .000) (see Figure 1).

The two separate repeated-measures ANOVA performed within each group of actions (human vs. non humans) confirmed the above results. While the effect of *Time* was significant for human actions [two levels, baseline (t1) vs. end of treatment (t10), F _(1,6)_ = 60.43; p = .000) revealing, as before, a significant improvement in the mean percentage of correct responses at end of treatment with respect to baseline performance (mean  = 66%, SEM  = 4 (T10) vs. mean  = 33%, SEM  = 3 (T1), p = .000), no significant effects were found between the two time conditions for non human actions (mean  = 38%, SEM  = 6 (T10) vs. mean  = 32%, SEM  = 5 (T1), p = .2). Moreover, for both group of actions (human and non human), no significant effect emerged between the different categories.

It might be argued that, since in the non human natural category we mixed both living (e.g, barking) and non-living (e.g., raining) actions, some possible dissociations in the patients' performance between the two subcategories might have been blinded not analyzing the data separately. Right now, a large amount of literature argues in favour of dissociable mechanisms in the production of living vs. non living things in aphasic patients [38]. In order to control for differences in the mean percentage of correct responses between the two classes, one separate repeated-measures 2×2 ANOVA was performed. Two within-subject factors were included: Time (two levels, baseline (T1) vs. end of treatment (T10)) and Condition (two levels: living vs. non living actions). The analysis revealed no significant effect between the two groups neither for Time [F_(1,6)_ = 0.73; p = .42], nor for Condition [F_(1,6)_ = 2.11; p = .20].

### Follow-ups

The analysis showed a significant effect of *Condition* (two levels, human vs. non-human, F _(1, 6)_  = 116.1; p = .000). The mean percentage of correct responses for human actions was significantly greater than for non human actions (mean  = 65%, SEM  = 4 (human actions) vs. mean  = 36%, SEM  = 4 (non human actions) p = .000). The interaction of *Time × Condition* (F _(3,18)_  = 4.93; p = .01) was also significant.

**Figure 2:**
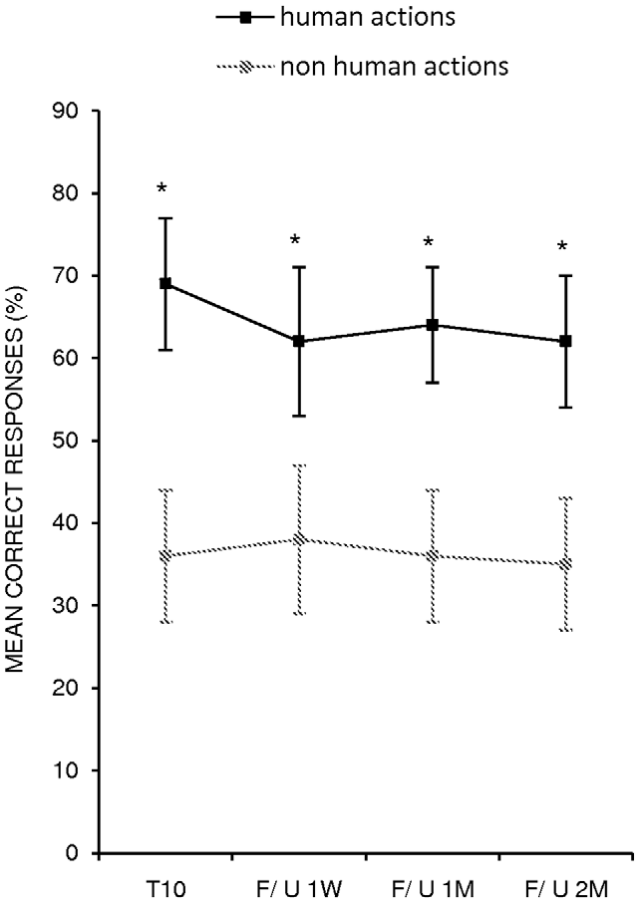
Mean percentage of correct responses for human and non human actions at end of treatment (T 10) vs. first follow-up (F/U, one week) vs. second follow-up (one month) vs. third follow-up (two months) (*p = .000). Error bars represent standard error of the mean (SEM).

The Scheffè post-hoc test revealed that the greater amount in the mean percentage of correctly named actions between human and non human actions observed at the end of treatment, was still present at the first (mean  = 62% (human actions), SEM  = 9 vs. mean  = 38%, SEM  = 9 (non human actions), p = .000), the second (mean  = 64% (human actions), SEM  = 7 vs. mean  = 36%, SEM  = 8 (non human actions), p = .000) and the third follow-up (mean  = 62% (human actions), SEM  = 8 vs. mean  = 35%, SEM  = 8 (non human actions), p = .000) (see Figure 2 and Table 2).

**Table 2:** Mean percentage of correct responses (± SEM).

	Human actions	Non-human actions
T1	36±8	29±6
T10	69±8	33±6
F/ U 1W	62±9	38±9
F/ U 1M	64±7	36±8
F/ U 2M	62±8	35±8

Note: T10  =  end of treatment; F/U 1W  =  follow-up one week; F/U 1M  =  follow-up one month; F/U 2M  =  follow-up two months.

### Transfer of verb treatment effects in the language examination tasks

Although only one patient made significant improvement in the verb naming task of the BADA test after the treatment (Pz 3, χ2 = 8.84, *p = .003*), we found significant differences in the description task of the Language Examination test (see Table 3). For six out of seven patients (one patient had already reached the maximum score before the treatment) chi square test indicated a significant difference in the percentage of correct responses before and after the therapy (p<.000).

**Table 3:** For each subject, the percentage of correct responses in the description task of the Language Examination are reported before and after the Observation Therapy.

Subject	Language Examination (before therapy)		Language Examination (after therapy)	
1	/Il micio gioca con la palla /Una signora che è seduta /, la mamma… con gatto e la palla, la palla con la palla giovane, indietro la mia mamma, qui il marito, /la bimba e la televisione/ /the cat is playing with the ball/A woman who is seated/the mother…with cat and the ball, the ball with the ball young, behind my mother, here the husband/the girl and the television/	30%	/Un bambino gioca con delle quadrucci /poi qui vicino c'è il padrone, non è il padrone, il bambino /il padre gioca, non gioca, legge il giornale /e sta vicino la bambina che sta guardando la televisione /Poi qui c'è un quadro, oddio come si chiama?/La signora si mette nel giornale e accarezza i ferri /poi il micio gioca con un … /Comunque questi sono moglie marito e due figli/ /A child is playing with some little squares/then near here there is the owner, is not the owner, the child/the father is playing, isn't playing, is reading the newspaper/and he is near the girl who is watching the television/Then here is a picture. Oh God, what is its name? The woman is putting herself in the newspaper and is gently touching the needles/then the cat is playing with one…/In any case, these are wife, husband and two kids/	80%
2	/Televisione, l'uomo, la donna maglia, libro, la finestra, bambino, bambina, la porta, tappeto. / Il gatto gioca./ /Television, the man, the woman, knit, book, the window, child, girl, the door, carpet/the cat is playing/	10%	/La donna mangia /uomo leggere /pampina televisione /....pampino cupo (cubo)...... /quattro (quadro)...libro, mobile....vaso..../.tue tue (tutti e due) siedere divano...tappeto/...filo...questa...libro....chiuse....chiudere divani tanda (tenda)....alberi......ah gatto bello.....filo/ /the woman is eating/man is reading/girl television/..child…cupo (block)…/four (picture)..book, furniture..vase/tue, tue (both) seat sofa..carpet/string this..book, close…close sofas tanda (curtain)…trees..ah beautiful cat string/	40%
3	/Ce sta na signora /quetta qua, gatto…poi questi qua lo so ma nun va. /there is a woman/this here, cat,..then these I know but I don't want/	0%	/Questa qua con la ragazza, gatto con la palla, /questa fa, la ragazza fa la….nun va… /sta a vedè, l'occhiali per vedè /questo qua (il bambino) sta a fa per mettere uno ad uno insieme/ /This here with the girl, cat with the ball/this does, the girl does the…doesn't work/ he is looking, the glasses to look/this here (the child) is putting one by one together/	30%
4	/Una donna fa la maglia /il gatto gioca con il gomitolo /la bambina guarda televisione /un uomo che legge il giornale /il bambino gioca. / /A woman is knitting/the cat is playing with the ball/the girl is watching the television/a man who is reading the newspaper/the child is playing/	95%	/Una donna fa la maglia /il gatto gioca con il gomitolo /la bambina guarda televisione /un uomo che legge il giornale /il bambino gioca con i cubi / /A woman is knitting/the cat is playing with the ball/the girl is watching the television/a man who is reading the newspaper/the child is playing with the blocks/	100%
5	/il gatto miao miao, /fa la….maglia /un papà legge le partite sul giornale /guarda la televisione /il maschietto gioca con i mattoncini / /the cat miao, miao/is knitting/a father is reading the football games on the newspaper/is watching the television/the boy is playing with the blocks/	60%	/Un gatto, no mangia, un gomitolo la che è, er gomitolo gioca /una donna … come l'uncinetto… /una bambina no parla… guarda uno schermo, una televisione /un uomo legge il giornale /un bambino gioca con i …. con un gioco…no gioco…cubi/ /a cat, doesn't eat, a ball that is a ball plays/a woman like the crochet/a girl doesn't speak, is watching a screen, a television/a man is reading the newspaper/a boy is playing with…a game, not game, blocks/	80%
6	/una casa, la lampada, un fiore, un gatto, un gattino, /l'uomo leggere giornale /televisione, la bimba telegiornale/ A house, a lamp, a flower, a cat, a little cat/the man read newspaper/television, the girl news/	10%	/l'uomo legge il giornale /la televisione è padre…la bambina /e l'uomo e prima due bambini antipatici,/la mamma a sedere /giocatto, il gatto e basta/ /the man is reading the newpaper/the television is father..the girl/and man and before two unpleasant children/the mother at seating/game, the cat and that's it/	40%
7	gatto, una donna, televisione, /…legge giornale /qua un bambino/ Cat, a woman, television/…is reading newspaper/here a child/	10%	/la signora fa….. /qui gatto che gioca /il signore legge il giornale /bambina tv /un bambino gioca con boh…non lo so /. /the woman does../here cat that is playing/the man is reading the newspaper/girl tv/a child plays with…don't know	60%

For all comparisons, chi square test indicated a significant difference before and after the therapy (p<.000).

## Discussion

The present study demonstrates that improvement in verb production in chronic aphasia after stroke can be achieved by intensive Action Observation Treatment over two consecutive weeks. Consistent with previous results [15], all patients showed a significant recovery of verb naming by observing video-clips of actions which still persisted after 2 months the treatment ended. This result allows us to confirm that Action Observation Treatment can be an useful strategy to enhance verb production. Most importantly, results clearly indicated that the amount of verb recovery was not equal across the two groups but increased significantly only for human actions.

The fact that the human brain exhibits such a great amount of plasticity and that language can improve late after stroke in such a short period of time may have important implications for future therapeutic interventions in aphasia. The present study suggests that the same basic principles relevant to enhance motor performance [21]–[24] may also be efficacious to improve language functions. These principles include 1) the use of massed practice for short time intervals that can be as effective as the use of long-term but less frequent training [39]–[41] 2) observation of actions that determines a strong impact on the recovery of verb production which persists over time 3) the fact that the therapy works only for actions belonging to the sensory-motor repertoire of the observer.

The choice to use such an intensive training was in accordance with recent proposals which suggest that, for stroke patients with motor deficits, intensive therapy over a short amount of time has greater impact on recovery than less intensive therapy over a long period of time [39]–[40]. Similar results were obtained in the language domain [41]. We found that such an intensive training exerts its influence when subjects are simply asked to observe video-clips of actions. As stated in the introduction, the embodied cognition viewpoint [12]–[13] suggests that the representation of a concept is composed not only of stored information about the features defining that concept, such as its typical form, colour and motion, but also of the motor movement associated with its use. Words whose retrieval is facilitated by gestures are more likely represented in sensory-motor features. The more a word is grounded in sensory-motor features, the more the actual execution of gestures will accompany its retrieval [11], [14]. Therefore, differently from previous reports, we have shown that not only action execution is an unnecessary prerequisite to enhance naming [9]–[11], but also that recovery might occur without verbal facilitation [6]–[8]. In agreement with a multimodal semantic representation proposal, we believe that together with the real execution of an action, the observation of the action directly interacts with the semantic system which enhances the activation of its corresponding sensory-motor representation. This activation serves as input at lexical level, and facilitates the retrieval of the word form. Although our data are strictly behavioural, we could speculate that the mechanism underlying this effect involves the Mirror Neuron System, which is equally active when actions are actually carried out and/or are simply observed [16]–[20]. Earlier studies have proposed that the Mirror Neuron System responds most robustly when watching familiar, executable actions made by conspecifics [25]. Brain regions associated with the Mirror System show stronger responses to human actions than to animal actions [25] and to actions made by robots [29]–[30]. These findings are consistent with the notion that observing actions with familiar kinematic features, which are within one's motor repertoire, result in greater Mirror Neuron involvement than observing less familiar actions. In line with most of the prior reports, we found that only human actions belonging to the sensory-motor experiential repertoire of the observer (e.g, dancing, eating) enhance verb recovery. These effects were consistently present even two months after the end of the treatment independently of the motor effectors (mouth, hand, foot, body) performing the action. We hypothesize that the observed familiar action exerts its influence at the semantic level because the sensory-motor representation, on which it is matched, produces an outcome that is known to the acting individual. Actions that are not part of the motor repertoire of the observer, such as natural (e.g, barking, raining) or mechanical actions (e.g., printing, digging) cannot make use of this matching process.

One final point regards a possible transfer of verb treatment effects in the language examination which would indicate a generalization of the recovery also in other tasks. Although only one patient made significant improvement in the verb naming task of the BADA test after the treatment, more interestingly, six out of seven patients showed a significant change in the description task with an increase in the use of verbs before and after the treatment. These different results might be due to the fact that while the verb naming task is administered using static and non-contextualised picture of actions, in the description task, verb production is more contextually-driven (e.g., the verb “to read” is presented in the context of a man who is reading a newspaper seated in an armchair) and therefore, in some ways, it better duplicates the situation used during the treatment (the video-clips presented were all contextually-driven).

In conclusion, our data clearly confirm that Action Observation Treatment is an useful rehabilitation strategy to promote a long-lasting recovery in verb production in aphasic patients. However, since the number of patients treated was small, the facilitation found between human and non human actions and their corresponding neural correlates deserve further investigations. We believe that these new findings open future directions for planning new therapeutic interventions for language rehabilitation.

## Supporting Information

Appendix S1 **List of human and non human videotaped actions presented in the language training.**
(DOC)
