# Supplementary material for: Walking but Not Barking Improves Verb Recovery: Implications for Action Observation Treatment in Aphasia Rehabilitation
Source: PLoS One. 2012 Jun 13;7(6):e38610. doi: 10.1371/journal.pone.0038610 (PMC3374821; doi:10.1371/journal.pone.0038610)
Supplement: Appendix S1 — List of human and non human videotaped actions presented in the language training. (DOC) [file pone.0038610.s001.doc]

**APPENDIX**

| **NON HUMAN ACTIONS** | **CATEGORY** |  | **NON HUMAN ACTIONS** | **CATEGORY** |
| --- | --- | --- | --- | --- |
| Esplodere (*to explode*) | mechanical |  | Abbaiare (*to bark*) | natural |
| Squillare (*to ring*) | mechanical |  | Cinquettare (*to tweet*) | natural |
| Annaffiare (*to water*) | mechanical |  | Volare (*to fly*) | natural |
| Crollare (*to collapse*) | mechanical |  | Strisciare (*to slither*) | natural |
| Stampare (*to print*) | mechanical |  | Beccare (*to peck*) | natural |
| Decollare (*to take off*) | mechanical |  | Fiorire (*to flower*) | natural |
| Sparare (*to shoot*) | mechanical |  | Scorrere (*to slide*) | natural |
| Frullare (*to blend)* | mechanical |  | Piovere (*to rain*) | natural |
| Pesare (*to weight*) | mechanical |  | Sorgere (*to rise*) | natural |
| Affondare (*to sink*) | mechanical |  | Tramontare (*to set*) | natural |
| Abbattere (*to put down)* | mechanical |  | Friggere (*to fry*) | natural |
| Scavare (*to dig*) | mechanical |  | Bollire (*to boil*) | natural |
| Sbattere (*to slam*) | mechanical |  | Bruciare (*to burn*) | natural |
| Sventolare (*to wave*) | mechanical |  | Sciogliere (*to melt*) | natural |
| Congelare (*to freeze*) | mechanical |  |  |  |
| Sollevare (*to pull up*) | mechanical |  |  |  |
| Abbassare (*to pull down*) | mechanical |  |  |  |
| Lavare (*to wash*) | mechanical |  |  |  |
| Fumare (*to smoke*) | mechanical |  |  |  |
| Caricare (*to charge*) | mechanical |  |  |  |
| Scaricare (*to upload*) | mechanical |  |  |  |
| Galleggiare (*to float*) | mechanical |  |  |  |
| Alzare (*to lift*) | mechanical |  |  |  |

| **HUMAN ACTIONS** | **CATEGORY** |  | **HUMAN ACTIONS** | **CATEGORY** |
| --- | --- | --- | --- | --- |
| Piegare (*to fold*) | Hand |  | Parare (*to save*) | Body |
| Toccare (*to touch*) | Hand |  | Sporcare (*to dirty*) | Body |
| Indicare (*to point*) | Hand |  | Sciare (*to sky*) | Body |
| Stirare (*to iron*) | Hand |  | Calciare (*to kick*) | Foot |
| Dipingere (*to paint*) | Hand |  | Camminare (*to walk*) | Foot |
| Infilare (*to thread*) | Hand |  | Pedalare (*to pedal)* | Foot |
| Tagliare (*to cut*) | Hand |  | Accavallare (*to cross*) | Foot |
| Cancellare (*to rub out)* | Hand |  | Pestare (*to stamp)* | Foot |
| Accendere (*to turn on)* | Hand |  | Calpestare (*to trample)* | Foot |
| Pettinare (*to comb)* | Hand |  | Pattinare (*to skate*) | Foot |
| Spremere (*to squeeze*) | Hand |  | Scendere (*to come down*) | Foot |
| Grattare (*to grate)* | Hand |  | Inciampare (*to stumble*) | Foot |
| Segare (*to saw)* | Hand |  | Spegnere (*to put out)* | Foot |
| Salutare (*to say hello)* | Hand |  | Frenare (*to slow down)* | Foot |
| Timbrare *(to stamp)* | Hand |  | Palleggiare (*to dribble*) | Foot |
| Buttare (*to throw away)* | Hand |  | Scavalcare *(to climb over)* | Foot |
| Cucire *(to sew)* | Hand |  | Pigiare (*to tread)* | Foot |
| Sbucciare (*to peel)* | Hand |  | Marciare (*to march*) | Foot |
| Massaggiare (*to massage)* | Hand |  | Sfilare (*to take off*) | Foot |
| Strizzare (*to squeeze out)* | Hand |  | Starnutire (*to sneeze)* | Mounth |
| Misurare (to measure) | Hand |  | Urlare (*to shout)* | Mounth |
| Sedersi *(to take a seat)* | Body |  | Baciare (*to kiss*) | Mounth |
| Spingere (*to push)* | Body |  | Masticare (*to chew)* | Mounth |
| Sudare *(to sweat)* | Body |  | Tossire *(to cough)* | Mounth |
| Colpire *(to shoot)* | Body |  | Bere (*to drink)* | Mounth |
| Seminare (*to sow)* | Body |  | Alitare (*to breath*) | Mounth |
| Dirigere *(to conduct)* | Body |  | Mordere (*to bite)* | Mounth |
| Nuotare (*to swim)* | Body |  | Soffiare (*to blow)* | Mounth |
| Cucinare (*to cook*) | Body |  | Gonfiare (*to blow up)* | Mounth |
| Rubare (*to steal)* | Body |  | Succhiare (*to suck)* | Mounth |
| Pescare (*to fish*) | Body |  | Ridere (*to laugh*) | Mounth |
| Apparecchiare (*to lay*) | Body |  | Assaggiare (*to taste)* | Mounth |
| Svenire (*to faint*) | Body |  | Cantare (*to sing*) | Mounth |
| Dondolare (*to lull*) | Body |  | Leccare (*to lick*) | Mounth |
| Ballare (*to dance*) | Body |  | Fischiare (*to whistle)* | Mounth |
| Annegare (*to drown*) | Body |  | Suggerire (*to whisper)* | Mounth |
| Tremare *(to tremble)* | Body |  | Sbadigliare (*to yawn*) | Mounth |
| Arrampicarsi (*to climb*) | Body |  | Sputare (*to spit)* | Mounth |
| Rompere (*to break*) | Body |  | Sbuffare (*to snort*) | Mounth |
